# Unveiling hidden neurodegeneration in isolated REM sleep behavior disorder through MRI microstructure and glymphatic flow

**DOI:** 10.1038/s41531-025-01193-8

**Published:** 2025-12-03

**Authors:** Silvia Basaia, Elisabetta Sarasso, Andrea Gardoni, Andrea Grassi, Alejandro Enrique Brivio, Sara Marelli, Roberta Balestrino, Lucia Zenere, Alessandra Castelnuovo, Massimo Malcangi, Elisa Canu, Luigi Ferini-Strambi, Federica Agosta, Massimo Filippi

**Affiliations:** 1 Neuroimaging Research Unit, Division of Neuroscience, IRCCS San Raffaele Scientific Institute, Milan, Italy; 2 Neurotech Hub, Vita-Salute San Raffaele University, Milan, Italy; 3 Department of Neuroscience, Rehabilitation, Ophthalmology, Genetics and Maternal Child Health, University of Genoa, Genoa, Italy; 4 Vita-Salute San Raffaele University, Milan, Italy; 5 Sleep Disorders Center, Division of Neuroscience, IRCCS San Raffaele Scientific Institute, Milan, Italy; 6 Neurology Unit, IRCCS San Raffaele Scientific Institute, Milan, Italy; 7 Neurorehabilitation Unit, IRCCS San Raffaele Scientific Institute, Milan, Italy; 8 Neurophysiology Service, IRCCS San Raffaele Scientific Institute, Milan, Italy

**Keywords:** Predictive markers, Translational research, Neurological disorders

## Abstract

This study aimed at: (1) assessing microstructural MRI and glymphatic flow alterations in isolated REM sleep behavioral disorder (iRBD) subjects relative to controls; (2) comparing sub-groups of iRBD patients with different levels of disease severity; and (3) studying the correlations between clinical alterations and MRI changes. 44 iRBD subjects and 52 controls underwent clinical and MRI evaluations. Gray and white-matter microstructural alterations were studied. Diffusion-tensor image analysis along the perivascular space (DTI-ALPS) index was obtained for the evaluation of glymphatic flow functionality. Cluster analysis was applied to divide iRBD patients in sub-groups. IRBD subjects showed worse sleep quality, reduced manual dexterity and gait alterations relative to controls. IRBD had alterations in the gray matter of fronto-parietal lobes and in the white matter of brainstem and frontal lobe, and a lower DTI-ALPS index relative to controls. Correlation analyses in the iRBD group showed that worse gray-matter microstructural alterations correlated with worse manual dexterity, lower peak turning velocity during dual-task mobility and worse sleep quality. Cluster analysis identified two clusters, one with worse clinical, neuropsychological, gait performances and DTI-ALPS index. The study detected early neurodegeneration in iRBD, subtle clinical deficits, microstructural gray/white-matter changes, and lower DTI-ALPS scores hinting at glymphatic dysfunction.

## Introduction

Rapid eye movement (REM) sleep behavior disorder (RBD) is a parasomnia characterized by excessive electromyographic (EMG) activity^1^, loss of muscle atonia of the skeletal muscle and vocalization during REM sleep^2,3^. Motor behaviors usually represent the violent content of the subject’s dreams^3^. Polysomnography (PSG) is the most reliable instrument to confirm the diagnosis of RBD^4^.

It is now well established that most patients with isolated RBD (iRBD) eventually progress to a neurodegenerative α-synucleinopathy such as Parkinson’s disease (PD), Lewy’s body dementia (LBD) or multiple system atrophy (MSA)^5,6^. However, the process of conversion varies significantly: it is still very difficult to predict which phenotype of Parkinsonism the patient will convert to, and the timing of phenoconversion and the trajectory of the developed disease over time are highly heterogeneous. Clinical assessments alone are insufficient for predicting disease development and progression; consequently, there is an urgent need for biomarkers capable of identifying individuals at high risk of neurodegeneration.

Growing attention has been given to glymphatic system dysfunction, a brain-wide clearance pathway that removes neurotoxic proteins such as α-synuclein and amyloid-β^7–9^. Evidence suggests that glymphatic activity is reduced in PD, correlating with motor and cognitive decline^8,10–14^, and recent studies have demonstrated that iRBD patients also exhibit reduced glymphatic function, as measured by diffusion tensor image analysis along the perivascular space (DTI-ALPS).

Diffusion tensor imaging (DTI) techniques have been widely employed to investigate microstructural brain alterations in iRBD, revealing subtle changes in white matter (WM) integrity across regions implicated in REM sleep regulation (e.g., thalamic radiation, substantia nigra), and sensorimotor processing, as well as associative tracts such as the temporal and occipital lobes and the fornix^15^. Despite its utility, DTI’s single-compartment model presents inherent limitations. To overcome DTI issues, a different diffusion MRI technique known as neurite orientation dispersion and density imaging (NODDI) has been introduced. NODDI refines diffusion MRI by modeling and separating signals from three tissue compartments: intra-neuritic water, extra-neuritic water, and cerebrospinal fluid. NODDI allows the estimation of neuronal density via the intracellular volume fraction (ICVF), neurites directional dispersion through the orientation dispersion index (ODI), and cerebrospinal fluid volume fraction through the isotropic water diffusion index (ISO)^16,17^. Another advantage of NODDI is the possibility to assess both WM and gray matter (GM) microstructural integrity. Moreover, NODDI can support the investigation of glymphatic function through integration with DTI-ALPS analysis.

Given the long prodromal phase of Parkinsonian syndromes and likely heterogeneity across iRBD, an alteration in the glymphatic system could play a role in this variability. Investigating iRBD subgroups stratified by varying levels of disease severity could provide valuable insights to verify this hypothesis. Thus, the aims of this study were to assess microstructural MRI and glymphatic flow alterations in iRBD subjects relative to controls, to compare subgroups of iRBD patients with different levels of disease severity to explore the variability in the neurodegenerative process, and to investigate the correlations between clinical changes, particularly gait alterations, and the observed MRI changes.

## Results

### Demographic and clinical data

Demographic and clinical variables are reported in Table 1 and in Supplementary Table 1. All groups were comparable in terms of age and education, while iRBD presented a higher number of males than controls (*p* = 0.02). IRBD subjects showed worse scores in most of the sleep questionnaires and presented motor and non-motor signs and symptoms, as shown by MDS-UPDRS scores (Table 1). iRBD also had alterations in the executive, memory and visuo-spatial cognitive domains (Table 1). Olfactory loss was the most frequent non-motor sign present in iRBD subjects (43%), followed by constipation (32%) and urinary dysfunction (27%) (Table 1).

**Table 1:** Sociodemographic and clinical characteristics of healthy controls and iRBD subjects

	HC (*N* = 52)	iRBD (*N* = 44)	*p* HC *vs* iRBD
Demographics			
Age [years]	62.83 ± 7.94 (40.72; 81.41)	65.46 ± 7.19 (51.88; 80.69)	0.20
Sex (M/F)	31/21	36/8	**0.02**
Disease duration [years]	/	6.55 ± 3.08 (5; 20)	/
Sleep			
RBDSQ	0 ± 0 (0; 0)	9.61 ± 1.65 (6; 12)	**<0.001**
PSQI	2.45 ± 1.88 (0; 7)	6.18 ± 3.13 (1; 13)	**<0.001**
ESS	3.58 ± 2.19 (0; 10)	5.20 ± 3.93 (0; 21)	0.11
Neurological evaluation			
MDS-UPDRS I	/	5.07 ± 3.35 (0; 13)	/
MDS-UPDRS II	/	0.66 ± 1.31 (0; 7)	/
MDS-UPDRS III [Bradykinesia]	/	3.02 ± 1.68 (1; 10)	/
MDS-UPDRS III [Resting tremor]	/	0.17 ± 0.771 (0; 4)	/
MDS-UPDRS III [Postural/Kinetic tremor]	/	0.63 ± 0.859 (0; 3)	/
MDS-UPDRS III [Rigidity]	/	1.51 ± 1.25 (0; 4)	/
MDS-UPDRS III [Gait/Balance]	/	0.24 ± 0.49 (0; 2)	/
MDS-UPDRS III [Facial expression]	/	0.27 ± 0.50 (0; 2)	/
MDS-UPDRS III	/	6.00 ± 2.60 (1; 11)	**/**
Neuropsychological assessment			
Digit span backward	4.90 ± 1.16 (3; 8)	4.45 ± 1.00 (3; 8)	0.55
Rey’s list immediate recall	51.03 ± 7.78 (36.40; 67)	44.39 ± 8.73 (25; 61)	**0.01**
Rey’s list delayed recall	11.35 ± 2.29 (7; 15)	8.82 ± 2.63 (3; 14)	**<0.001**
Raven’s progressive matrices	33.08 ± 2.45 (25; 36)	29.64 ± 4.76 (16; 36)	**<0.001**
Attentive matrices	54.78 ± 5.04 (39;60)	52.48 ± 6.12 (29; 60)	0.07
Token test	34.50 ± 1.33 (31; 36)	33.89 ± 1.70 (28; 36)	0.24
Benson’s figure copy	15.70 ± 0.76 (13; 17)	14.57 ± 1.39 (11; 16)	**<0.001**
Benson’s figure recall	11.30 ± 3.00 (4; 17)	10.07 ± 3.04 (2; 16)	0.07
Motor functional assessment			
9HPT [s]	21.15 ± 3.25 (12.50; 26.85)	23.65 ± 3.07 (16.50; 30.96)	**0.01**
10MWT – CS [s]	8.03 ± 1.08 (5.99; 10.77)	8.48 ± 1.18 (6.18; 12.24)	0.20
5TSTS [s]	10.51 ± 2.33 (7; 18)	11.66 ± 2.48 (6.68; 16.20)	0.08
DT cost TUG [%]	6.75 ± 12.41 (**–**9.81; 49.07)	14.38 ± 13.41 (**–**3.59; 54.51)	0.06
Non-motor symptoms			
Olfactory loss [Y/N]	/	19/25	/
Constipation [Y/N]	/	14/30	/
Urinary dysfunction [Y/N]	/	12/32	/
Orthostatic hypotension [Y/N]	/	1/43	/
Mood			
Depressive symptoms [Y/N]	5/46	3/41	1.00

Values are means ± standard deviations (minimum; maximum). Categorical variables are reported as frequency. *p* values refer to ANOVA adjusted for age and sex or chi-square test for categorical variables Bonferroni corrected for number of groups (*p* < 0.05).

*5TSTS* 5-Time sit-to-stand, *9HPT* 9-hole pegboard test, *10MWT* 10-meter walking test, *CS* comfortable speed, *DT* dual task, *ESS* Epworth Sleepiness Scale, *HC* healthy controls, *iRBD* subjects with isolated REM sleep behavior disorder, *M/F* male/female, *MDS-UPDRS-I or II or III* Movement Disorder Society Unified Parkinson’s Disease Rating Scale part I or part II or part III, *p* *p*-value, *PSQI* Pittsburgh Sleep Quality Index, *RBDSQ* REM Sleep Behavior Disorder Screening Questionnaire, *s* seconds, *TUG* timed up-and-go.

Bold is for significant values.

iRBD subjects showed the presence of some motor alterations, with a higher time in the Nine-Hole-Peg-Test (9HPT) (*p* = 0.01), more steps in 10-meter-walking-test (10MWT) both at normal and maximal speed and a trend toward (*p* = 0.06) a higher dual task (DT) cost in the Timed up and Go test (TUG) (Table 1; Supplementary Table 1). Moreover, several spatio-temporal gait parameters such as arm swing amplitude, gait pattern velocity, asymmetry, and stride length were altered in TUG and 4-meter walking test (4MWT) both with and without dual-task (Supplementary Table 1**)**.

### Tract-based spatial statistics (TBSS) results

**Fig. 1:**
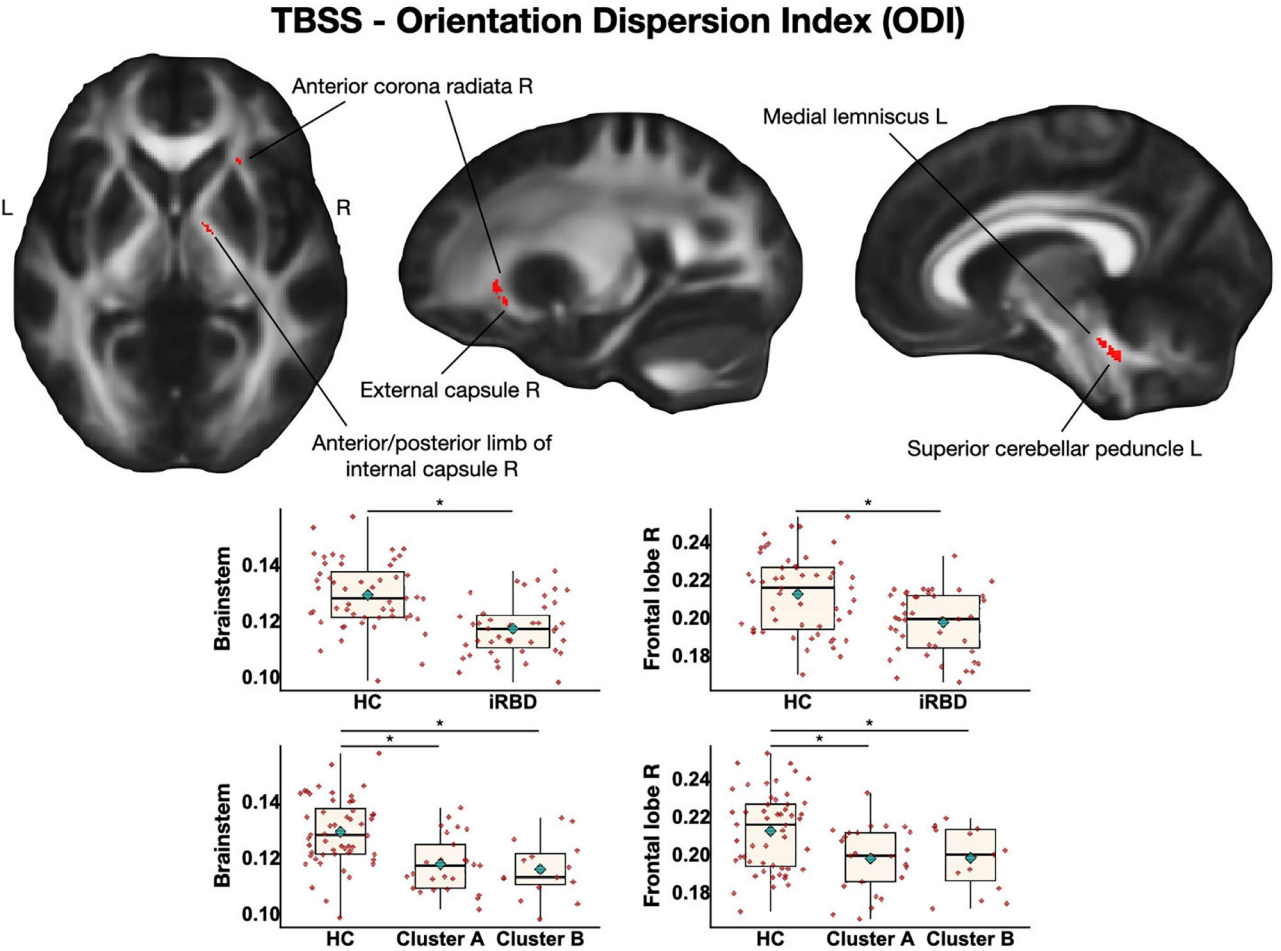
Tract-based spatial statistics results in patients with isolated REM sleep behavioral disorder (iRBD) versus healthy controls. Decreased orientation dispersion index (ODI) is shown in red. Results are overlaid on the axial, coronal and sagittal sections of the Montreal Neurological Institute standard brain, and displayed at *p* < 0.05 corrected for multiple comparisons. Box plot of white matter values from each ROI of the USCL atlas are shown for each group (healthy controls, iRBD patients together or separated by cluster). The black horizontal line in each box plot represents the mean, light-blue diamond indicates the median, while the red diamonds represent individual subjects, and whiskers represent the minimum and maximum values. HC healthy controls, iRBD subjects with isolated REM sleep behavior disorder, L left, R right, TBSS Tract-Based Spatial Statistic.

Significant results are shown in Fig. 1 and described below. iRBD subjects showed only a reduction of ODI relative to controls, with a trend toward statistical significance (*p* < 0.08), in left superior cerebellar peduncle and medial lemniscus, anterior corona radiata and right anterior and posterior limbs of the internal capsule and the external capsule. Considering that the result is approaching statistical significance, we obtained the mean WM values from each ROI of the USCL atlas. In this case, the analysis showed a significant reduced ODI in the brainstem (*p* < 0.001) and in the right part of the frontal lobe (*p* = 0.002) in iRBD subjects compared to controls. No significant results were found in fractional anisotropy (FA) and ICVF measures on WM maps.

### Gray-matter-based spatial statistics (GBSS) results

**Fig. 2:**
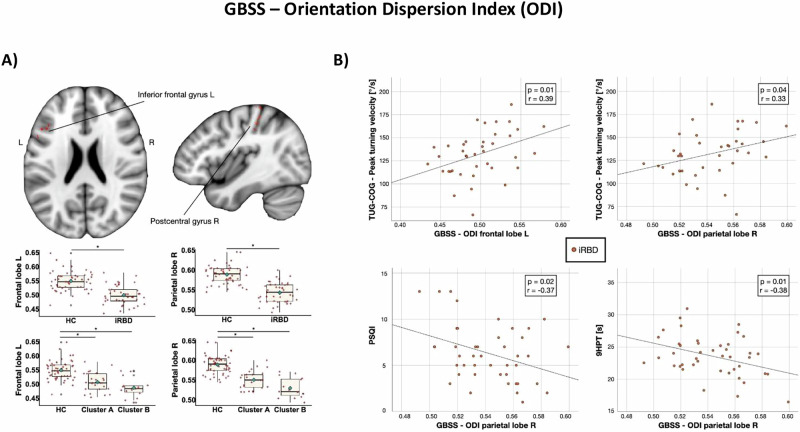
Gray matter-based spatial statistics in isolated REM sleep behavioral disorder (iRBD): group differences and associations with clinical measures. **A** Gray-matter based spatial statistics results in patients with isolated REM sleep behavioral disorder (iRBD) versus healthy controls. Decreased orientation dispersion index (ODI) is shown in red. Results are overlaid on the axial and sagittal sections of the Montreal Neurological Institute standard brain, and displayed at *p* < 0.05 corrected for multiple comparisons. Box plot of gray matter values from each ROI of the USCL atlas are shown for each group (healthy controls, iRBD patients together or separated by cluster). The black horizontal line in each box plot represents the mean, light-blue diamond indicates the median, while the red diamonds represent individual subjects, and whiskers represent the minimum and maximum values. **B** Correlation plot between gray matter values from each ROI of the USCL atlas and clinical data in iRBD subjects. 10MWT 10-meter walking test, GBSS Gray-matter-Based Spatial Statistics, HC healthy controls, iRBD subjects with isolated REM sleep behavior disorder, L left, ODI orientation dispersion index, PSQI Pittsburgh Sleep Quality Index, R right, s seconds, TUG timed up-and-go.

Significant results are shown in Fig. 2A and described below. In GM maps, significant decreased ODI was found in the right postcentral gyrus and in the left inferior frontal gyrus in iRBD patients relative to controls (*p* < 0.05). Considering the USCL atlas, the analysis confirmed a significant reduction of ODI in the right frontal (*p* < 0.001) and in the left parietal (*p* < 0.001) lobes. No significant results were found in FA and ICVF measures on GM maps.

### DTI–ALPS index

**Fig. 3:**
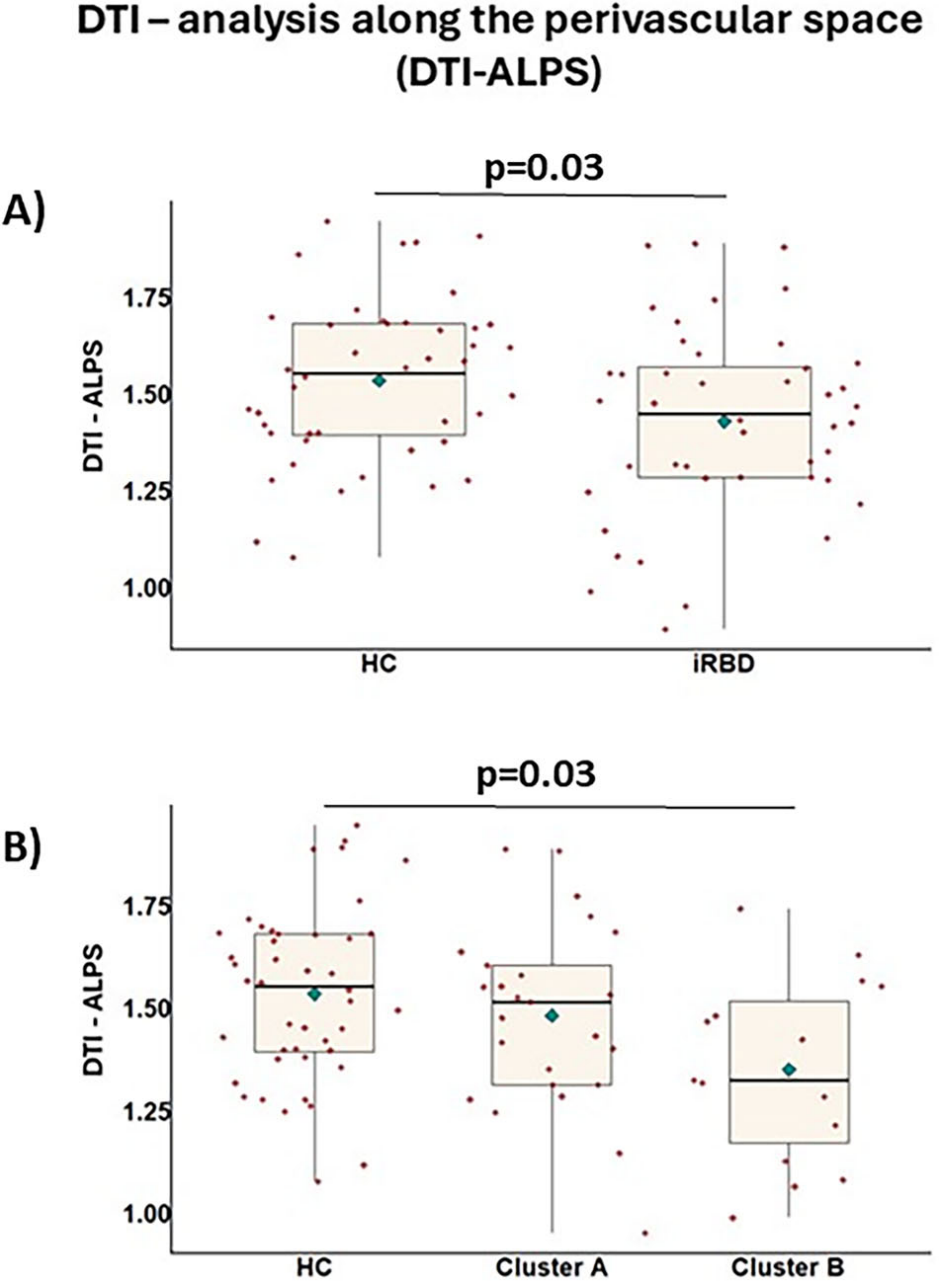
DTI-ALPS results in iRBD patients and healthy controls. Box plot for DTI-ALPS index in healthy controls, iRBD patients together (**A**) or separated by cluster (**B**). The black horizontal line in each box plot represents the mean, light-blue diamond indicates the median, while the red diamonds represent individual subjects, and whiskers represent the minimum and maximum values. Two-sided *p-*value < 0.05 was considered for statistical significance. HC healthy controls, iRBD subjects with isolated REM sleep behavior disorder.

Compared to controls, iRBD subjects showed significantly lower DTI-ALPS index (*p* = 0.03) (Fig. 3A).

### Correlation results

In iRBD subjects, a reduced GM ODI in the left frontal lobe correlated with a reduced peak turning velocity during TUG-COG (*r* = 0.39, *p* = 0.01; Fig. 2B) and UPDRS-III (*r* = –0.41, *p* = 0.02; Fig. 2B). Moreover, a lower GM ODI value in the right parietal lobe correlated with a higher score in PSQI (*r* = –0.37, *p* = 0.02; Fig. 2B) and a higher time to perform the 9HPT (*r* = –0.38, *p* = 0.01; Fig. 2B).

### Clustering analysis results

According to the data-driven clustering analysis, the highest silhouette index was estimated with respect of two identified iRBD clusters: Cluster A consisting of 25 subjects and Cluster B consisting of 15 subjects; four subjects were included in neither group during the clustering process (Table 2 and Supplementary Table 2).

**Table 2:** Sociodemographic and clinical characteristics of healthy controls and the two RBD clusters

	HC (*N* = 52)	iRBD Cluster A (*N* = 25)	iRBD Cluster B (*N* = 15)	*p* HC *vs* Cluster A	*p* HC *vs* Cluster B	*p* Cluster A *vs* Cluster B
Demographics						
Age [years]	62.83 ± 7.94 (40.72; 81.41)	62.38 ± 6.46 (51.88; 73.82)	68.90 ± 6.56 (58.67; 80.69)	1.00	**0.02**	**0.02**
Sex [M/F]	31/21	23/2	10/5	**0.004**	0.66	**0.04**
Disease duration [years]	/	5.84 ± 1.55 (5; 10)	7.27 ± 4.28 (5; 20)	/	/	0.27
Sleep						
RBDSQ	0 ± 0 (0; 0)	9.24 ± 1.64 (6; 12)	10.20 ± 1.47 (7; 12)	**<0.001**	**<0.001**	**0.03**
PSQI	2.45 ± 1.88 (0; 7)	5.56 ± 2.92 (1; 12)	7.60 ± 3.18 (3; 13)	**<0.001**	**<0.001**	0.14
ESS	3.58 ± 2.19 (0; 10)	4.16 ± 2.30 (0; 10)	7.47 ± 5.24 (1; 21)	1.00	**<0.001**	**<0.001**
Neurological evaluation						
MDS-UPDRS I	/	3.72 ± 3.27 (0; 13)	7.40 ± 2.47 (4; 13)	/	/	**0.002**
MDS-UPDRS II	/	0.64 ± 1.5 (0; 7)	0.80 ± 1.15 (0; 4)	/	/	1.00
MDS-UPDRS III [Bradykinesia]	/	2.96 ± 1.84 (1; 10)	3.27 ± 1.39 (1; 6)	/	/	1.00
MDS-UPDRS III [Resting tremor]	/	0.28 ± 0.98 (0; 4)	0 ± 0 (0; 0)	/	/	1.00
MDS-UPDRS III [Postural/Kinetic tremor]	/	0.64 ± 0.76 (0; 2)	0.67 ± 1.05 (0; 3)	/	/	1.00
MDS-UPDRS III [Rigidity]	/	1.56 ± 1.29 (0; 4)	1.47 ± 1.25 (0; 4)	/	/	1.00
MDS-UPDRS III [Gait/Balance]	/	0.08 ± 0.28 (0; 1)	0.47 ± 0.64 (0; 2)	/	/	0.32
MDS-UPDRS III [Facial expression]	/	0.28 ± 0.46 (0; 1)	0.13 ± 0.35 (0; 1)	/	/	1.00
MDS-UPDRS III	/	5.88 ± 2.73 (1; 11)	6.27 ± 2.52 (3; 11)	/	/	1.00
Neuropsychological assessment						
Digit span backward	4.90 ± 1.16 (3; 8)	4.72 ± 1.10 (3; 8)	4.07 ± 0.80 (3; 6)	1.00	0.23	0.39
Rey’s list immediate recall	51.03 ± 7.78 (36.40; 67)	46.64 ± 7.20 (33; 61)	41.27 ± 9.86 (25; 57)	0.60	**<0.001**	0.07
Rey’s list delayed recall	11.35 ± 2.29 (7; 15)	9.40 ± 2.40 (4; 14)	7.93 ± 3.03 (3; 13)	0.07	**<0.001**	0.17
Raven’s progressive matrices	33.08 ± 2.45 (25; 36)	31.40 ± 3.37 (24; 36)	26.40 ± 5.69 (16; 34)	0.17	**<0.001**	**<0.001**
Attentive matrices	54.78 ± 5.04 (39;60)	54.60 ± 4.86 (46; 60)	49.13 ± 7.05 (29; 59)	1.00	**0.01**	0.07
Token test	34.50 ± 1.33 (31; 36)	34.52 ± 1.17 (32; 36)	32.87 ± 2.07 (28; 36)	1.00	**0.01**	**0.02**
Benson’s figure copy	15.70 ± 0.76 (13; 17)	14.96 ± 1.02 (13; 16)	14.00 ± 1.56 (11; 16)	**0.02**	**<0.001**	0.07
Benson’s figure recall	11.30 ± 3.00 (4; 17)	10.80 ± 2.36 (7; 16)	9.07 ± 3.37 (3; 14)	1.00	**0.048**	0.36
Motor functional assessment						
9HPT	21.15 ± 3.25 (12.50; 26.85)	22.55 ± 3.10 (16.50; 29.51)	25.47 ± 2.45 (22.10; 30.96)	0.26	**<0.001**	0.09
10MWT – CS [s]	8.03 ± 1.08 (5.99; 10.77)	8.23 ± 0.88 (6.86; 10.30)	8.79 ± 1.60 (6.18; 12.24)	1.00	0.17	0.72
5TSTS [s]	10.51 ± 2.33 (7; 18)	11.28 ± 2.54 (6.68; 16.20)	12.13 ± 1.95 (9.46; 14.88)	0.38	0.14	1.00
DT cost TUG [%]	6.75 ± 12.41 (**-**9.81; 49.07)	9.69 ± 8.54 (-1.35; 25.73)	21.93 ± 15.59 (-2.38; 54.51)	1.00	**0.003**	**0.045**
Non-motor symptoms						
Olfactory Loss [Y/N]	/	10/15	7/8	/	/	1.00
Constipation [Y/N]	/	2/23	9/6	/	/	**<0.001**
Urinary dysfunction [Y/N]	/	2/23	10/5	/	/	**<0.001**
Orthostatic hypotension [Y/N]	/	1/24	0/15	/	/	0.86
Mood						
Depressive symptoms [Y/N]	5/46	2/23	1/14	1.00	1.00	1.00

Values are means ± standard deviations (minimum; maximum). Categorical variables are reported as frequency. *p* values refer to ANOVA adjusted for age and sex or chi-square test for categorical variables Bonferroni corrected for number of groups (*p* < 0.05).

*5TSTS* 5-Time sit-to-stand, *9HPT* 9-hole pegboard test, *10MWT* 10-meter walking test, *CS* comfortable speed, *DT* dual task, *ESS* Epworth Sleepiness Scale, *HC* healthy controls, *iRBD* subjects with isolated REM sleep behavior disorder, *M/F* male/female, *MDS-UPDRS-I or II or III* Movement Disorder Society Unified Parkinson’s Disease Rating Scale part I or part II or part III, *p* *p*-value, *PSQI* Pittsburgh Sleep Quality Index, *RBDSQ* REM Sleep Behavior Disorder Screening Questionnaire, *s* seconds, *TUG* timed up-and-go.

Bold is for significant values.

*Demographic and clinical data*. The two clusters were different from each other regarding demographic and clinical characteristics, with Cluster B being significantly older and showing worse scores in RBD screening questionnaire (RBDSQ), Epworth Sleepiness Scale (ESS), MDS-UPDRS I, executive functions, global cognition, TUG-COG and DT cost of TUG (Table 2 and Supplementary Table 2). Considering non-motor symptoms, constipation and urinary dysfunction were more common in Cluster B (60% and 66.6%) than in Cluster A (both 8%). Considering gait analysis data, Cluster B relative to Cluster A had higher arm swing amplitude asymmetry during 4MWT and stride time asymmetry during 4MWT-COG, together with worse stride length both during TUG and TUG-COG (Supplementary Table 2). Generally, cluster B showed more significant differences relative to controls compared to Cluster A both in motor and non-motor features (Table 2 and Supplementary Table 2).

*MRI results*. In TBSS analysis, values in the brainstem (*p* < 0.001) and right frontal lobe (*p* = 0.001) appeared to be reduced in both clusters compared to controls, with no differences between clusters (Fig. 2). Considering GBSS results in the USCL ROIs, both clusters exhibited lower GM ODI in the left frontal (*p* < 0.001) and right parietal lobes (*p* < 0.001; Fig. 2A).

Relative to controls, only Cluster B exhibited reduced DTI-ALPS scores with worse glymphatic flow functionality (*p* = 0.03; Fig. 3B).

## Discussion

This study is a continuation of a recently published work from our research group^18^ and aimed at deepening previous findings with brain diffusion MRI hallmarks of PSG-confirmed iRBD. In this work, we focused on the WM and GM microstructural characteristics and glymphatic flow changes and on the correlations between the latter MRI patterns and subtle clinical alterations that might represent initial neurodegenerative processes as previously suggested^5,6,19^. The analysis of diffusion MRI data using NODDI sequence provided an insight on the glymphatic system and on both WM and GM microstructural alterations in iRBD subjects. We also conducted a cluster analysis in order to divide subjects in different groups according to the presence of motor, non-motor and cognitive signs and to test if different clinical clusters also showed different gait analysis and diffusion MRI parameters.

Focusing on clinical findings in the whole sample, as expected, iRBD subjects had worse sleep quality, and subtle motor signs of Parkinsonism, as reflected by the MDS-UPDRS III scores and analyzing the gait pattern with the optoelectronic system. These findings suggest an early impairment of motor circuits^20,21^. Despite these motor alterations, their impact on daily life remained minimal, as indicated by low scores on the MDS-UPDRS II, which evaluates functional autonomy in everyday activities. This suggests that while subclinical motor signs are present, they have not yet resulted in overt disability.

Regarding the non-motor symptoms, many patients already presented olfactory dysfunction (hyposmia or anosmia), autonomic alterations such as urinary disturbances and constipation, as well as subtle cognitive changes, particularly affecting executive functions, episodic memory, and visuospatial abilities. These signs were detected through the MDS-UPDRS Part I and specific neuropsychological batteries. The combined presence of non-motor symptoms is well-documented as part of the prodromal phenotype of PD, with olfactory dysfunction being one of the earliest and most reliable biomarkers of neurodegeneration^22^.

Our results obtained through TBSS revealed a reduced WM ODI in the whole sample of iRBD subjects compared to controls, particularly in the superior cerebellar peduncle, medial lemniscus, anterior corona radiata and internal and external capsule. According to our knowledge, no study applied NODDI to investigate microstructural alterations in iRBD patients, therefore, our results provide new insights in the neural correlates of the disease. Existing literature on diffusion imaging in iRBD mainly used standard diffusivity metrics such as FA, mean diffusivity and axial diffusivity to provide information on pathophysiological mechanisms with heterogeneous results^15,23^. Overall, these studies reported microstructural abnormalities in regions related to sleep, sensorimotor and cognitive processing in iRBD^15^, as well as in structures associated with neurodegenerative pathology in PD^23–25^. Interpretation of ODI alterations is not simple and univocal; however, we can speculate that a decrease ODI could be the result of reduced neurite complexity, possibly due to dendritic loss^26^. In our sample, microstructural alterations of the superior cerebellar peduncle may suggest a structural disconnection due to damaged WM fibers connecting supratentorial brain structures with the cerebellum that, as previously suggested, is involved in gait alterations typical of PD and MSA^27–30^. We also found changes in the internal capsule that is a subcortical structure with high concentration of motor and sensory fibers projecting to and from cortical areas. The posterior limb plays a fundamental role in movement regulation with pyramidal and extrapyramidal motor pathways passing through^31^. The anterior corona radiata, which was also involved in our sample, includes projection from prefrontal cortex that plays a role in the cognitive control of gait^32^. Therefore, our results showed how different WM tracts involved in motor control and cognition can present microstructural alteration in patients with iRBD, supporting existing literature suggesting how WM alterations might precede GM atrophy in prodromal and early PD,^18^ phases^33^.

Despite GM alteration seems not to be prominent in iRBD and early PD^18^ GBSS analysis provided the possibility to study the microstructure of GM that might show alterations before seeing an evident GM atrophy. Our GBSS results showed GM microstructural changes in the inferior frontal and postcentral gyri in iRBD patients relative to controls. The inferior frontal gyrus is part of the prefrontal cortex that plays a pivotal role in cognitive control of tasks especially when they require both motor and cognitive involvement^34^. Postcentral gyrus is engaged in motor-sensory feedback and previous studies reported a functional decline of this brain area in PD patients with iRBD, which can represent a deficit in sensorimotor integration^35^. Therefore, patients with iRBD showed initial microstructural alteration in GM of brain areas involved in motor control. Interestingly, we found significant correlation between these alterations and clinical data in the iRBD group. A reduced GM ODI in the parietal lobe correlated with a slower peak turning velocity during TUG-COG and a worse performance at the 9HPT supporting the presence of an initial alteration of sensorimotor control in iRBD patients^18^.

Notably, group differences emerged for ODI but not for FA or ICVF. A reduction in ODI indicates lower angular dispersion and thus greater fiber coherence. In regions with pronounced crossing architecture (e.g., corona radiata, internal/external capsules, superior cerebellar peduncle, medial lemniscus), this pattern is biologically consistent with a selective loss of one fiber population or pruning of collateral projections, which increases directional coherence without necessarily reducing neurite density at early stages. Such early geometric reorganization can precede detectable changes in ICVF. Methodologically, FA conflates effects of microstructural damage and orientation dispersion; a decrease in dispersion tends to increase FA and may offset FA reductions due to subtle damage, yielding null group differences.

DTI-ALPS analysis showed a reduced functionality in the glymphatic system in iRBD subjects compared to controls. Previous works suggested a relationship between sleep and glymphatic function, in particular a lower excretion of α-synuclein from brain parenchyma may contribute to the development of Parkinsonism in patients with iRBD^12–14,36^. Therefore, as previously suggested^8^, DTI-ALPS may become a useful tool in the study of prodromal Parkinsonisms and conversion from iRBD to manifest diseases^8^. Of course, in order to speculate about possible biomarkers of specific phenoconversion, longitudinal studies are fundamental to develop accurate predictive models^37^.

Our clustering analysis resulted in two clusters, likely representing subjects with a worse disease course (Cluster B) and subjects with a relatively more benign progression (Cluster A). Indeed, Cluster B showed worse sleep scores, cognitive state and motor signs, and a worse gait pattern with an increased upper and lower limb asymmetry of movement and a reduced stride length both during single-task and dual-task walking. We can speculate that Cluster B presents more Parkinsonian-like characteristics, even if we cannot guess which type of Parkinsonism will develop and after how many years. Notably, Cluster B also showed a more reduced GM ODI in the parietal lobe. This result should be considered preliminary as it did not survive correction for number of groups. Interestingly, DTI-ALPS index was significantly lower relative to controls only in Cluster B. Given the globally worse condition of patients in Cluster B, this finding is consistent with the results of a recent study that reported how the phenoconversion risk in iRBD patients increased with a decreased DTI-ALPS index^8^.

This study is not without limitations. The sample size was relatively small. Considering the exploratory nature of our study and the wide number of clinical variables, we presented clinical results corrected for the number of group comparisons to limit type 2 error. Moreover, this study employed a cross-sectional design, which limits the possibility to track disease progression, therefore, future longitudinal studies are needed to provide clearer information about the role of diffusion MRI, and particularly NODDI, to predict phenoconversion to clinically defined Parkinsonisms. Another limitation of our study concerns the TBSS analysis. Although trends toward differences between iRBD and controls were observed, no statistically significant results emerged (*p* < 0.08). These findings should therefore be interpreted with caution, as they may reflect false positives rather than true group effects. Further studies with larger cohorts and complementary diffusion analysis approaches are needed to clarify whether subtle microstructural alterations exist. In addition, it is important to acknowledge the intrinsic limitations of the DTI-ALPS metric. As an indirect and model-dependent measure, DTI-ALPS provides only an approximation of glymphatic function and may not capture its full complexity. Therefore, our results should be interpreted within the framework of these methodological constraints.

This study highlights early neurodegenerative changes in individuals with iRBD, evidenced by subtle motor, non-motor, and cognitive impairments and microstructural alterations in GM and WM. Reduced DTI-ALPS scores indicated glymphatic dysfunction, potentially related to pathological protein accumulation. Cluster analysis suggested glymphatic impairment as a possible marker of disease progression. Correlation findings support the role of both microstructural and glymphatic changes in subtle clinical manifestations of iRBD. Longitudinal studies are warranted to define the potential role of diffusion MRI in predicting iRBD progression to Parkinsonism.

## Methods

### Standard protocol approvals, registrations, and patient consents

The ethical standards committee on human experimentation of IRCCS San Raffaele Scientific Institute (Milan, Italy) approved the study protocol and all participants provided written informed consent prior to study inclusion.

### Subjects

Forty-four patients with PSG-confirmed diagnosis of iRBD according to the International Classification of Sleep Disorders (ICSD)-3 criteria^38^ were recruited at the Sleep Disorders Center, Division of Neuroscience, IRCCS San Raffaele Scientific Institute (Milan, Italy). Of these, 38 iRBD patients had already been included in our previous study^18^. Fifty-two age- and sex-matched healthy controls were recruited among non-consanguineous relatives, institute personnel and by word of mouth. Inclusion criteria for patients and healthy controls were: (i) right handedness; (ii) native Italian speakers; (iii) age 50–75 years; (iv) Mini Mental State Examination (MMSE) score ≥24; (v) oral and written informed consent to study participation. Exclusion criteria were: (i) secondary forms of RBD on the basis of historical data, neurologic examination, and brain MRI findings; (ii) history of (other) systemic, neurologic, psychiatric diseases, head injury, cardiovascular events; (iii) brain damage at routine MRI, including lacunae and extensive cerebrovascular disorders; (iv) alcohol and/or psychotropic drugs abuse.

All participants underwent motor functional, gait analysis and cognitive evaluations, and brain MRI, as previously described^18^. Patients with iRBD also underwent PSG and neurological evaluations. Sleep disorders in controls were assessed using the Pittsburgh Sleep Quality Index (PSQI)^39^, RBDSQ^40^, Epworth Sleepiness Scale (ESS)^41^, Insomnia Severity Index (ISI)^42^, STOP-Bang Questionnaire^43^, and International Restless Legs Study Group Severity Rating Scale (IRLS)^44^.

### Neurological, motor functional, gait analysis and neuropsychological evaluations

An experienced neurologist assessed sleep quality and motor/non-motor impairments in iRBD patients with the MDS-UPDRS^45^ and the Non-Motor Symptoms Scale (NMS)^46^. Olfaction was assessed through clinical interview and the olfactory loss item of the NMS (>4 meaning reduced olfaction). An experienced neuropsychologist performed a comprehensive cognitive assessment investigating global cognition, memory, attention and executive functions, language, visuospatial abilities, and mood^18^. An experienced physiotherapist administered the 9HPT, the Five-Time-Sit-To-Stand (5TSTS), and the 10MWT.

A six-camera SMART-DX7000 (BTS Bioengineering, Italy) optoelectronic system was used to obtain spatio-temporal gait parameters^18^. Specifically, we acquired the TUG in order to study the turning phase of gait and 4MWT to study straight walking parameters. Both TUG and 4MWT were performed also associated with a cognitive dual-task (TUG-COG and 4MWT-COG). Asymmetry between right and left side was calculated from upper and lower limb parameters as: $\frac{{right}-{left}}{\max ({right;left})}\times 100$. We calculated the dual-task cost (DTcost) as: $\frac{{dual\; task}-{single\; task}}{{single\; task}}\times 100$.

### MRI acquisition

All patients and controls underwent brain MRI scans on a 3.0 Tesla MRI scanner (Ingenia CX, Philips Medical Systems, Best, The Netherlands) at San Raffaele Hospital, Milan. The following brain MRI sequences were obtained from all participants: 3D T1-weighted turbo field echo (TFE) (TR = 7 ms; TE = 3.2 ms; flip angle = 9 [degrees]; 204 contiguous sagittal slices with voxel size = 1 × 1 × 1 mm, matrix size = 256 × 240, FOV = 256 × 240 mm^2^); 3D fluid-attenuated inversion recovery (FLAIR) (TR = 4800 ms; TE = 267 ms; TI = 1650 ms; ETL = 167; NEX = 2; 192 contiguous sagittal slices with voxel size = 0.89 × 0.89 × 1 mm, matrix size = 256 × 256, FOV = 256 × 256 mm^2^); 3D T2 (TR = 2500 ms; TE = 330 ms; ETL = 117; NEX = 1; 192 contiguous sagittal slices with voxel size = 0.89 × 0.89 × 1 mm, matrix size = 256 × 258, FOV = 256 × 256 mm^2^); 3D susceptibility weighted image (SWI): FOV = 230 × 189, pixel size = 0.60 × 0.60 × 2 mm, 135 slices, 1 mm thick (slice oversampling), matrix = 384×313, TR = 31 ms, TE = 7.2 ms, FA = 17; and axial pulsed-gradient spin echo (PGSE) single shot DW EPI sequence: shells at *b* value = 700/1000/2855 s/mm^2^ along 6/30/60 non-collinear directions and 10 *b* = 0 volumes were acquired (TR = 5900 ms, TE = 78 ms, voxel size = 2.14 × 2.69 mm, 56 slices, 2.3 mm thick, matrix size = 112 × 85, FOV = 240 × 233 mm^2^).

### Pre-processing of diffusion-weighted imaging

Preprocessing of diffusion-weighted data included skull-stripping and correction for head motions by aligning the volumes to the first B0 volume, as well as susceptibility-induced field and eddy currents distortion correction, using the tools implemented in the FMRIB software library (FSL, version 5.0.9)^47^. The diffusion tensor was estimated by linear regression using only the low-b shell (*b* < 1000 s/mm²) with FSL’s dtifit, from which FA maps were derived. For the NODDI model, ICVF and ODI maps were computed using the NODDI Matlab Toolbox with default settings (http://www.nitrc.org/projects/noddi_toolbox).

### TBSS and GBSS analysis

Voxel-wise diffusion tensor MRI analysis was performed using TBSS tool implemented in FSL (version 5.0.9) (http://www.fmrib.ox.ac.uk/fsl/fdt/index.html) to obtain FA, ICVF and ODI diffusion skeletonized WM maps. Moreover, GBSS technique was applied to assess voxel-wise differences in GM microstructure^48,49^. For each subject, FA maps obtained from the previous DWI processing steps were aligned to the FMRIB58 template included in FSL, using non-linear registration (fsl FNIRT tools). A mean FA image was calculated, averaging all the aligned images in the common space, and the result was thinned to obtain a skeleton representing only the WM tracts common to all the subjects. The FA skeletonized maps were thresholded to exclude voxels below the intensity of 0.2. The FA maps of each individual were projected to this skeleton mask. ICVF and ODI were also projected to the same skeleton mask, using the tbss_non_FA tool in FSL^50^. For GBSS analysis, 3DT1, T2 and DWI scans of each patient were registered through linear and non-linear transformation to the MNI152 space (2 mm resolution), using FSL FLIRT and FNIRT tools. The resulting warping matrices were subsequently applied to the GM probability maps (obtained from VBM segmentation) and NODDI-derived ICVF and ODI maps, to align all images to the MNI152 common space. An average GM probability map was calculated using fslmaths and skeletonized. For each patient, local voxels with the greatest GM probability were projected to the GM skeleton. The GM skeletonized map was thresholded, keeping voxels higher than 0.65 in more than 75% of the subjects. For each subject, individual diffusion metrics from ICVF and ODI maps were projected on the thresholded skeleton from the voxels with the greatest GM probability obtained from the previous step. The remaining missing voxels from the skeleton mask were filled with the average of the surrounding voxels on the skeleton, weighted by closeness with a Gaussian Kernel (*σ* = 2 mm). Considering TBSS analysis, three different 4D WM images (with all the subjects) were created including FA, ICVF and ODI measures. In GBSS analysis, the process returns 4D skeletonized maps of ICVF and ODI on the GM. To perform subsequent regional analysis, the 4D WM/GM images were then co-registered to USCLobes brain atlas (http://brainsuite.org/usclobes-description), as previously described^51^. For each subject, mean values of skeletonized FA (only from WM maps), ICVF and ODI from WM/GM maps were obtained for each region of interest (ROI) of the USCLobes brain atlas (bilateral frontal, parietal, temporal, occipital lobes, bilateral insula, bilateral cingulate, brainstem, cerebellum, corpus callosum).

### DTI-ALPS index quantification

DTI-ALPS is an index that evaluates the diffusivity along the direction of the perivascular space compared with those of projection fibers and association fibers on a slice at the level of the lateral ventricle body.

For each participant, the FA color map, the 3D FLAIR image, were registered onto the SWI space using the magnitude of the first echo of the SWI sequence as a reference image. All transformations were visually inspected to ensure proper coregistration.

Using the venous vessel map obtained from SWI images, we selected, for each participant, 3 contiguous axial slices where veins run perpendicular to the lateral ventricles to accurately define brain regions where veins, and thus perivascular spaces, are aligned along the *x*-axis^52^.

On a color-coded FA map, we placed a 3 × 3 × 3 mm cubical ROI in the area of the projection fibers and the area of the association fibers in the left hemisphere. For each ROI, we calculated the diffusivity in the directions of the *x*-axis, *y*-axis, and *z*-axis. We calculated the DTI-ALPS index in order to evaluate the activity of the glymphatic system in individual cases. This index is provided by the ratio of two sets of diffusivity value which are perpendicular to dominant fibers in the tissue, that is the ratio of mean of *x*-axis diffusivity in the area of projection fibers (Dxproj) and *x*-axis diffusivity in the area of association fibers (Dxassoc) to the mean of *y*-axis diffusivity in the area of projection fibers (Dyproj) and *z*-axis diffusivity in the area of association fibers (Dzaccoc) as follows^52^:

1 $$DTI-ALPSindex=\frac{{mean}({Dxproj},{Dxassoc})}{{mean}({Dyproj},{Dzassoc})}$$

A higher DTI-ALPS index indicates better glymphatic function.

### Statistical analysis

Demographic and clinical data were reported as means and standard deviations or frequencies and percentages for continuous and categorical variables, respectively.

Demographic, clinical and MRI data (mean values of FA, ICVF, and ODI of different ROIs obtained from WM and GM maps and DTI-ALPS index) were compared between iRBD and controls and between iRBD sub-groups (see clustering analysis below). Analysis of variance (ANOVA) with post hoc test was used for continuous variables (correcting *p* values for multiple comparisons using the Bonferroni method) and Chi-squared test for categorical variables. Two-sided *p*-value < 0.05 was considered for statistical significance. Statistical analysis was performed using the R software.

Partial correlation analysis was performed using Pearson’s correlation coefficient (R) between MRI data (DTI-ALPS index and diffusion-derived measures in both TBSS and GBSS) and clinical data, adjusting for age, and sex (*p* < 0.05). *P* values were corrected for multiple comparisons using the Bonferroni method.

Voxelwise statistics on TBSS and GBSS skeletonized maps, comparing the different groups, was performed using a non-parametric, permutation-based, inference tool (“randomise”, part of FSL), using age, sex, and education as covariates with 5000 permutations. The results were corrected for multiple comparisons with the threshold-free cluster enhancement (TFCE) and displayed with a *p* < 0.05.

### Clustering analysis

Cluster analysis based on k-medoids method for data partitioning was applied on iRBD subjects using the Gower distance calculated for different baseline data on demographic/general clinical information (age, sex, and disease duration), sleep variables (RBDSQ, PSQI and ESS scores), motor symptoms/signs (MDS-UPDRS I-II, MDS-UPDRS-III divided in bradykinesia, resting tremor, kinetic/postural tremor, rigidity, gait/balance and facial expression subscores, 9HPT, 10MWT, 5TSTS, DT cost of TUG), cognition and mood (digit span backward, immediate recall of Rey’s list, delayed recall of Rey’s list, Raven’s progressive matrices, attentive matrices, token test, copy of Benson’s figure, recall of Benson’s figure, depression), and the presence of other non-motor manifestations (olfactory loss, constipation, urinary dysfunction, orthostatic hypotension, depression). We performed both internal and external validation of the clustering solution. For internal validation, the number of clusters was determined using the silhouette coefficient and the Calinski–Harabasz index, both of which supported a two-cluster solution. For external validation, we assessed cluster robustness via a bootstrap stability analysis with 1000 resamples (sampling with replacement). For each bootstrap sample, we computed the adjusted Rand index (ARI) against the clustering from the full dataset.

## Supplementary information

Supplementary Information

## Data Availability

The dataset used and analyzed during the current study will be made available by the corresponding author upon request to qualified researchers (i.e., affiliated to a university or research institution/hospital).
