# Supplementary material for: Unveiling hidden neurodegeneration in isolated REM sleep behavior disorder through MRI microstructure and glymphatic flow
Source: NPJ Parkinsons Dis. 2025 Dec 3;11:346. doi: 10.1038/s41531-025-01193-8 (PMC12675625; doi:10.1038/s41531-025-01193-8)
Supplement: Supplementary file 1 — Supplementary Information [file 41531_2025_1193_MOESM1_ESM.docx]

**Supplementary Table 1.** Clinical, neuropsychological and gait analysis characteristics of healthy controls and iRBD subjects*.*

|  | **HC (N=52)** | **iRBD (N=44)** | **p HC *vs* iRBD** |
| --- | --- | --- | --- |
| **NEUROPSYCHOLOGICAL ASSESSMENT** | | | |
| **MMSE** | 29.28 ± 0.88  (26; 30) | 28.70 ± 1.34  (24; 30) | 0.19 |
| **Corsi block tapping test** | 5.68 ± 1.08  (4; 7) | 5.34 ± 0.94  (3; 7) | 0.33 |
| **TMT B-A** | 50.16 ± 16.62  (20.36; 84.78) | 57.16 ± 28.68  (12; 138) | 0.56 |
| **Phonemic verbal fluency** | 42.77 ± 9.44  (26; 64) | 38.23 ± 12.55  (14; 74) | 0.30 |
| **Semantic verbal fluency** | 53.16 ± 8.25  (39; 68) | 49.45 ± 10.06  (26; 69) | 0.39 |
| **MOTOR FUNCTIONAL ASSESSMENT** | | | |
| **10MWT – CS NS** | 13.96 ± 1.28  (11; 17) | 14.64 ± 1.50  (11.67; 19) | **0.02** |
| **10MWT – MS [s]** | 6.01 ± 0.61  (4.75; 7.05) | 6.58 ± 2.01  (4.60; 18.50) | 0.17 |
| **10MWT – MS NS** | 12.17 ± 1.11  (11; 15.33) | 12.72 ± 1.44  (10.30; 16) | **0.02** |
| **TUG [s]** | 9.47 ± 1.79  (6.80; 13.94) | 9.89 ± 1.61  (7.40; 14.75) | 0.53 |
| **TUG – COG [s]** | 10.13 ± 2.43  (6.76; 16.51) | 11.31 ± 2.28  (8.02; 18.16) | 0.09 |
| **GAIT ANALYSIS** | | | |
| **Arm swing amplitude asy (4MWT) [%]** | 1.01 ± 0.93  (0.05; 3.72) | 3.10 ± 2.49  (0.02; 9.81) | **<0.001** |
| **Peak arm swing vel**  **(4MWT) [°/s]** | 102.12 ± 39.34  (46.62; 220.57) | 84.99 ± 23.80  (35.46; 156.98) | **0.03** |
| **Mean arm swing vel**  **(4MWT) [°/s]** | 59.76 ± 27.85  (19.37; 148.36) | 48.13 ± 17.63  (16.27; 100.83) | **0.04** |
| **Mean walking speed**  **(4MWT-COG) [m/s]** | 1.57 ± 0.27  (0.89; 2.26) | 1.43 ± 0.20  (0.98; 1.94) | **0.03** |
| **Stride length**  **(4MWT-COG) [%h]** | 84.97 ± 9.69  (62.16; 102.23) | 80.39 ± 7.86  (65.40; 98.23) | 0.06 |
| **Stride time asymmetry (4MWT-COG) [%]** | 0.49 ± 0.37  (0.01; 1.52) | 0.70 ± 0.60  (0.01; 2.81) | 0.16 |
| **Peak arm swing vel**  **(4MWT-COG) [°/s]** | 106.73 ± 39.39  (42.08; 227.63) | 88.14 ± 21.30  (48.40; 143.74) | **0.01** |
| **Mean arm swing vel**  **(4MWT-COG) [°/s]** | 61.25 ± 28.32  (18.26; 151.66) | 47.95 ± 15.81  (20.55; 93.88) | **0.01** |
| **Stride length**  **(TUG) [%h]** | 73.74 ± 7.80  (58.27; 89.06) | 70.12 ± 7.62  (56.89; 85.05) | 0.10 |
| **Peak turning vel**  **(TUG) [°/s]** | 159.99 ± 26.08  (110.89; 215.53) | 144.24 ± 19.46  (96.03; 182.98) | **0.01** |
| **Mean turning vel**  **(TUG) [m/s]** | 1.18 ± 0.20  (0.77; 1.62) | 1.09 ± 0.16  (0.71; 1.36) | **0.04** |
| **Stride length**  **(TUG-COG) [%h]** | 72.42 ± 9.30  (49.38; 88.78) | 67.18 ± 7.77  (51.16; 84.22) | **0.02** |
| **Peak turning vel**  **(TUG-COG) [°/s]** | 153.55 ± 31.46  (79.93; 227.09) | 131.15 ± 25.43  (66.44; 186.08) | **0.002** |
| **Mean turning vel**  **(TUG-COG) [m/s]** | 1.12 ± 0.24  (0.62; 1.71) | 1.01 ± 0.18  (0.60; 1.37) | **0.03** |

*Values are means ± standard deviations (minimum; maximum). Categorical variables are reported as frequency. p values refer to ANOVA adjusted for age and sex or chi-square test for categorical variables Bonferroni corrected for number of groups (p < 0.05).* ***Abbreviations:*** *4MWT (-COG) = 4-meters walking test (with cognitive dual-task); asy = asymmetry; h = height; HC = healthy controls; iRBD = subjects with isolated REM sleep behavior disorder; L/R = left/right; m = meters; s = seconds; TMT B-A = Trail Making Test part B minus part A; TUG (-COG) = Timed up-and-go (with cognitive dual-task); vel = velocity; Y/N = yes/no.*

**Supplementary Table 2**. Clinical, neuropsychological and gait analysis characteristics of healthy controls and the two clusters.

|  | **HC**  **(N=52)** | **iRBD**  **Cluster A (N=25)** | **iRBD**  **Cluster B (N=15)** | **p**  **HC *vs* Cluster A** | **p**  **HC *vs* Cluster B** | **p**  **Cluster A *vs* Cluster B** |
| --- | --- | --- | --- | --- | --- | --- |
| **NEUROPSYCHOLOGICAL ASSESSMENT** | | | | | | |
| **MMSE** | 29.28 ± 0.88  (26; 30) | 29.20 ± 0.96  (27; 30) | 28.07 ± 1.67  (24; 30) | 1.00 | **0.003** | **0.01** |
| **Corsi block tapping test** | 5.68 ± 1.08  (4; 7) | 5.60 ± 0.87  (4; 7) | 4.73 ± 0.70  (3; 6) | 1.00 | **0.02** | 0.08 |
| **TMT B-A** | 50.16 ± 16.62  (20.36; 84.78) | 46.28 ± 22.11  (12; 94) | 71.53 ± 26.02  (26; 121) | 1.00 | **0.03** | **0.02** |
| **Phonemic verbal fluency** | 42.77 ± 9.44  (26; 64) | 41.76 ± 13.45  (15; 74) | 31.73 ± 10.17  (14; 51) | 1.00 | **0.02** | 0.05 |
| **Semantic verbal fluency** | 53.16 ± 8.25  (39; 68) | 53.12 ± 8.81  (35; 69) | 43.60 ± 10.01  (26; 62) | 1.00 | **0.01** | **0.02** |
| **MOTOR FUNCTIONAL ASSESSMENT** | | | | | | |
| **10MWT – CS NS** | 13.96 ± 1.28  (11; 17) | 14.12 ± 1.26  (11.67; 17.67) | 15.40 ± 1.62  (12.33; 19) | 0.65 | **0.01** | 0.15 |
| **10MWT – MS [s]** | 6.01 ± 0.61  (4.75; 7.05) | 6.39 ± 2.60  (4.60; 18.50) | 6.85 ± 0.73 (5.37; 7.99) | 0.31 | 0.83 | 1.00 |
| **10MWT – MS NS** | 12.17 ± 1.11  (11; 15.33) | 12.07 ± 1.16  (10.30; 16) | 13.73 ± 1.26  (12; 16) | 1.00 | **<0.001** | **0.003** |
| **TUG [s]** | 9.47 ± 1.79  (6.80; 13.94) | 9.46 ± 1.16  (7.40; 12.34) | 10.52 ±1.78  (8.53; 14.75) | 1.00 | 0.11 | 0.22 |
| **TUG – COG [s]** | 10.13 ± 2.43  (6.76; 16.51) | 10.37 ± 1.46  (8.02; 14.27) | 12.84 ± 2.77  (8.80; 18.16) | 1.00 | **0.003** | **0.02** |
| **GAIT ANALYSIS** | | | | | | |
| **Arm swing amplitude asy**  **(4MWT) [%]** | 1.01 ± 0.93  (0.05; 3.72) | 2.33 ± 2.14  (0.02; 7.20) | 4.39 ± 2.49  (0.24; 9.81) | 0.04 | **<0.001** | **0.01** |
| **Peak arm swing velocity (4MWT) [°/s]** | 102.12 ± 39.34  (46.62; 220.57) | 83.29 ± 21.03  (53.52; 129.72) | 89.79 ± 30.08  (35.46; 156.98) | 0.05 | 0.63 | 1.00 |
| **Mean arm swing velocity (4MWT) [°/s]** | 59.76 ± 27.85  (19.37; 148.36) | 47.01 ± 16.01  (26.37; 83.96) | 51.48 ± 21.74  (16.27; 100.83) | 0.08 | 0.70 | 1.00 |
| **Mean walking speed**  **(4MWT-COG) [m/s]** | 1.57 ± 0.27  (0.89; 2.26) | 1.49 ± 0.20  (1.06; 1.94) | 1.34 ± 0.17  (0.98; 1.56) | 0.38 | **0.02** | 0.45 |
| **Stride length**  **(4MWT-COG) [%h]** | 84.97 ± 9.69  (62.16; 102.23) | 82.62 ± 7.86  (65.40; 98.23) | 76.32 ± 6.95  (66.47; 87.78) | 0.72 | **0.02** | 0.27 |
| **Stride time asy**  **(4MWT-COG) [%]** | 0.49 ± 0.37  (0.01; 1.52) | 0.50 ± 0.49  (0.01; 1.83) | 1.07 ± 0.65  (0.10; 2.81) | 1.00 | **<0.001** | **<0.001** |
| **Peak arm swing velocity (4MWT-COG) [°/s]** | 106.73 ± 39.39  (42.08; 227.63) | 88.11 ± 21.37  (60; 143.74) | 88.90 ± 23.34  (48.40; 143.57) | 0.05 | 0.14 | 1.00 |
| **Mean arm swing vel**  **(4MWT-COG) [°/s]** | 61.25 ± 28.32  (18.26; 151.66) | 47.44 ± 16.07  (27.92; 93.88) | 48.72 ± 17.02  (20.55; 83.59) | **0.048** | 0.15 | 1.00 |
| **Stride length**  **(TUG) [%h]** | 73.74 ± 7.80  (58.27; 89.06) | 72.87 ± 7.33  (58.25; 85.05) | 65.41 ± 6.05  (56.89; 76.56) | 1.00 | **0.01** | **0.045** |
| **Peak turning vel**  **(TUG) [°/s]** | 159.99 ± 26.08  (110.89; 215.53) | 150.64 ± 15.42  (116.92; 182.98) | 136.08 ± 21.09  (96.03; 171.87) | 0.20 | **0.01** | 0.33 |
| **Mean turning vel**  **(TUG) [m/s]** | 1.18 ± 0.20  (0.77; 1.62) | 1.14 ± 0.14  (0.82; 1.36) | 1.01 ± 0.14  (0.71; 1.20) | 0.55 | **0.02** | 0.28 |
| **Stride length**  **(TUG-COG) [%h]** | 72.42 ± 9.30  (49.38; 88.78) | 70.25 ± 6.86  (58.33; 84.22) | 61.68 ± 6.63  (51.16; 71.14) | 0.72 | **<0.001** | **0.03** |
| **Peak turning vel**  **(TUG-COG) [°/s]** | 153.55 ± 31.46  (79.93; 227.09) | 141.58 ± 21.47  (108.76; 186.08) | 115.01 ± 22.78  (66.44; 142.92) | 0.18 | **<0.001** | 0.07 |
| **Mean turning vel**  **(TUG-COG) [m/s]** | 1.12 ± 0.24  (0.62; 1.71) | 1.07 ± 0.15  (0.75; 1.37) | 0.90 ± 0.17  (0.60; 1.11) | 0.55 | **0.01** | 0.16 |

*Values are means ± standard deviations (minimum; maximum). Categorical variables are reported as frequency. p values refer to ANOVA adjusted for age and sex or chi-square test for categorical variables Bonferroni corrected for number of groups (p < 0.05).* ***Abbreviations:*** *4MWT (-COG) = 4-meters walking test (with cognitive dual-task); asy = asymmetry; h = height; HC = healthy controls; iRBD = subjects with isolated REM sleep behavior disorder; L/R = left/right; m = meters; MMSE = Mini-Mental State Examination; s = seconds; TMT B-A = Trail Making Test part B minus part A; TUG (-COG) = Timed up-and-go (with cognitive dual-task); vel = velocity; Y/N = yes/no.*
